# Brain death-induced cytokine release is not associated with primary graft dysfunction: a cohort study

**DOI:** 10.5935/0103-507X.20190009

**Published:** 2019

**Authors:** Tatiana Helena Rech, Geisiane Custódio, Leonardo Viliano Kroth, Sabrina Frighetto Henrich, Édison Moraes Rodrigues Filho, Daisy Crispim, Cristiane Bauermann Leitão

**Affiliations:** 1 Programa de Pós-Graduação em Ciências Médicas: Endocrinologia, Universidade Federal do Rio Grande do Sul - Porto Alegre (RS), Brasil.; 2 Unidade de Terapia Intensiva, Hospital de Clínicas de Porto Alegre, Universidade Federal do Rio Grande do Sul - Porto Alegre (RS), Brasil.; 3 Unidade de Transplante Renal, Hospital São Lucas - Porto Alegre (RS), Brasil.; 4 Unidade de Terapia Intensiva, Hospital São Vicente de Paulo - Passo Fundo (RS), Brasil.; 5 Unidade de Terapia Intensiva, Hospital Dom Vicente Scherer - Porto Alegre (RS), Brasil.; 6 Divisão de Endocrinologia, Hospital de Clínicas de Porto Alegre, Universidade Federal do Rio Grande do Sul - Porto Alegre (RS), Brasil.

**Keywords:** Brain death, Inflammation, Cytokines, Primary graft dysfunction, Deceased donor, Transplantation

## Abstract

**Objective:**

To examine the association between donor plasma cytokine levels and the development of primary graft dysfunction of organs transplanted from deceased donors.

**Methods:**

Seventeen deceased donors and the respective 47 transplant recipients were prospectively included in the study. Recipients were divided into two groups: group 1, patients who developed primary graft dysfunction; and group 2, patients who did not develop primary graft dysfunction. Donor plasma levels of TNF, IL-6, IL-1β, and IFN-γ assessed by ELISA were compared between groups.

**Results:**

Sixty-nine organs were retrieved, and 48 transplants were performed. Donor plasma cytokine levels did not differ between groups (in pg/mL): TNF, group 1: 10.8 (4.3 - 30.8) *versus* group 2: 8.7 (4.1 - 33.1), p = 0.63; IL-6, group 1: 1617.8 (106.7 - 5361.7) *versus* group 2: 922.9 (161.7 - 5361.7), p = 0.56; IL-1β, group 1: 0.1 (0.1 - 126.1) *versus* group 2: 0.1 (0.1 - 243.6), p = 0.60; and IFN-γ, group 1: 0.03 (0.02 - 0.2) *versus* group 2: 0.03 (0.02 - 0.1), p = 0.93). Similar findings were obtained when kidney transplants were analyzed separately.

**Conclusion:**

In this sample of transplant recipients, deceased donor plasma cytokines TNF, IL-6, IL-1β, and IFN-γ were not associated with the development of primary graft dysfunction.

## INTRODUCTION

Brain death (BD) leads to an inflammatory condition associated with adverse outcomes in organ transplantation in experimental^(^^1,2^^)^ and clinical settings.^(^^3^^)^ Kidney grafts from HLA-mismatched living donors are known to perform better than kidneys from deceased donors.^(^^3^^)^ Brain death-induced inflammatory activity is characterized by the upregulation of plasma cytokines, as demonstrated in previous studies by our group^(^^4-6^^)^ and, together with other important factors, plays a role in the development of primary graft dysfunction (PGD). This inflammatory trigger adversely affects organ function and is one of the possible pathways associated with clinical outcomes of transplants.^(^^7,8^^)^ Kusaka et al. compared rat models of BD to controls and showed a dense inflammatory infiltrate in the glomerular tubules of brain-dead animals.^(^^9^^)^ In line with this finding, Contreras et al. demonstrated that BD-induced inflammatory activity had an adverse impact on islet function in rats, increasing apoptosis in beta cells.^(^^10^^)^

Primary graft dysfunction is a common complication of deceased donor organ transplantation. This dysfunction is associated with increased risk of graft loss in the first 36 months of follow-up, as well with increased length of hospital stay^(^^11^^)^ and increased costs.^(^^12^^)^ The association between BD and PGD, however, is not fully understood. It is supposed that by activating the inflammatory cascade, BD can be a key component of ischemia-reperfusion injury, an effect that might be even more pronounced in organs from expanded criteria donors. Interestingly, there is an association for the development of PGD across different organs transplanted from the same donor.^(^^13^^)^

The present study was designed to examine the association between donor plasma tumour necrosis factor (TNF), interleukin-6 (IL-6), interleukin-1β (IL-1β), and interferon-gamma (IFN-γ) and the development of PGD of organs transplanted from deceased donors.

## METHODS

### Deceased donors and transplant recipients

The study protocol was approved by the Ethics Committee (number 13-060) at *Hospital de Clínicas de Porto Alegre*, in the city of Porto Alegre, and three other participating transplant centers: *Hospital São Lucas* and *Hospital Dom Vicente Scherer*, both in Porto Alegre, and *Hospital São Vicente de Paulo*, in Passo Fundo, in agreement with the Helsinki declaration of 1975. All institutions are in Rio Grande do Sul, the southernmost state of Brazil. Informed consent was obtained from the patients' legal representatives. Brain death was assessed independently by two physicians and was based on the following criteria: coma with complete unresponsiveness, absence of brain stem reflexes, apnea test, and confirmatory test with absence of cerebral blood flow according to the Brazilian law.^(^^14^^)^ From November 2010 to December 2011, brain-dead patients older than 18 years admitted to the intensive care unit at *Hospital de Clínicas de Porto Alegre* were prospectively included in the study after the first clinical examination consistent with BD. Blood samples were collected at study entry. Organ recipients were identified from a crossover list provided by the regional organ distribution center. Clinical and laboratory data were recorded for brain-dead donors and transplant recipients.

Allograft dysfunction was defined as follows: (1) renal: requirement for dialysis during the first week after transplant;^(^^15^^)^ (2) liver: primary nonfunction during the first week after transplant leading to retransplantation or death or initial poor function characterized by aspartate aminotransferase > 2,000IU/L, serum bilirubin > 10mg/dL, or prothrombin time >16 seconds within 2 - 7 days after transplant;^(^^16^^)^ (3) lung: development of severe hypoxemia, lung edema and radiographic opacities compatible with acute respiratory distress syndrome during the first 3 days after transplant;^(^^17^^)^ and (4) heart: need for mechanical support, such as external ventricular assist device, aortic counterpulsation pump, and extracorporeal membrane oxygenation, in the first 3 days after transplant or retransplantation/death during the first 30 days.^(^^18^^)^

### Plasma TNF, IL-6, IL-1β, and IFN-γ quantification

A 20mL whole blood sample was collected in a silicone-coated tube (Vacutainer^®^) for each brain-dead donor and centrifuged at 2,500g for 10 minutes at 4ºC. Plasma was separated and immediately stored at -80ºC until analysis. Circulating levels of TNF, IL-6, IL-1β, and IFN-γ were assessed by enzyme-linked immunosorbent assay (ELISA) using commercially available kits with primary polyclonal antibodies following the manufacturer's instructions (detection levels: TNF, 0.7 - 518pg/mL; IL-6: 20 - 5000pg/mL; IL-1β: 0.35 - 1166pg/mL; and IFN-γ: 0.03 - 30pg/mL; Biosource Europe S.A., Nivelles, Belgium).

### Statistical analysis

Categorical variables were expressed as percentages. Quantitative data were expressed as the mean and standard deviation (SD) if normally distributed. Variables with skewed distributions were log-transformed before analysis and expressed as median and minimum-maximum. Groups were compared using Student's *t* test, chi-square test or Fisher's exact test, as appropriate. Spearman's rank correlation was used to assess correlations between different quantitative variables. A sample size of 32 organ transplant recipients (16 patients with PGD and 16 patients without PGD) was required to detect a difference of at least one SD in TNF log,^(^^4^^)^ considering a power of 80% and an alpha-error of 5%. Values were considered to be statistically significant if p < 0.05. All statistical analyses were performed using Statistical Package for Social Science (SPSS), version 18.0 (Chicago, IL).

## RESULTS

A total of 69 organs were retrieved from 17 deceased donors (a mean of 4.05 organs per donor): 34 kidneys, 17 pancreases, 13 livers, four lungs, and one heart. All pancreases were used for research purposes, and one liver and two lungs were discarded due to technical problems. Forty-eight transplants were performed in 47 patients (one patient underwent a simultaneous liver-kidney transplant). The characteristics of 38 transplant recipients (data were not available for nine patients) and 17 deceased donors included in the study are summarized in table 1. Briefly, 70% of donors were men with a mean ± SD age of 54 ± 11 years; stroke was the leading cause of BD (76.5%) followed by anoxic encephalopathy (23.5%). All deceased donors required vasopressor support, and sepsis was present in 41%.

**Table 1:** Baseline characteristics of deceased donors and organ transplant recipients

Characteristics	All transplant recipients (n = 38)	With graft dysfunction (n = 20)	Without graft dysfunction (n = 18)	p value
Donor characteristics				
Age (years)	54 ± 11	56 ± 11.2	50 ± 11.4	0.09
Plasma sodium (mEq/L)	158 ± 9.7	156 ± 8.2	158 ± 12.1	0.54
Final creatinine (mg/dL)	1.2 ± 0.5	1.2 ± 0.6	1.2 ± 0.4	0.92
Duration of ventilatory support (days)	3 (1 - 10)	4 (1 - 10)	2 (1 - 10)	0.07
LOS before BD diagnosis (days)	4 (1 - 14)	5.5 (1 - 14)	2.5 (1 - 13)	0.09
Recipient characteristics				
Age (years)	53 ± 14	56 ± 10	49 ± 17	0.15
Male	30 (79)	17 (44.7)	13 (34.3)	0.43
Transplanted organ	28 kidneys, 9 livers, 1 heart	17 kidneys, 3 livers, 0 heart	11 kidneys, 6 livers, 1 heart	0.14*
Cold ischemia time (hours)	17 ± 7.6	19 ± 6.8	14 ± 8.1	0.07
LOS after transplantation (days)	23 (1 - 92)	25 (1 - 92)	22 (1 - 86)	0.74
Graft survival at 12 months	30 (81)	16 (44)	14 (37)	0.69
Patient survival at 12 months	25 (67.5)	11 (29.7)	14 (37.8)	0.29
Total mortality	6 (16.2)	3 (8.1)	3 (8.1)	1.0

LOS - length of stay; BD - brain death. p values refer to patients with graft dysfunction vs patients without graft dysfunction.

* p value refers to kidney transplants. Results are presented as mean ± standard deviation, median and minimum - maximum, or n (%).

**Figure 1:**
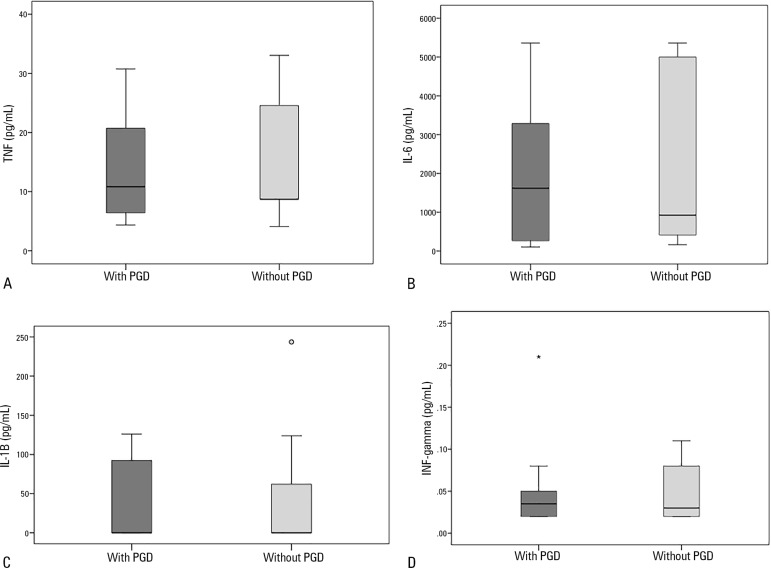
Deceased donor plasma cytokine levels determined by ELISA in transplant recipients with and without primary graft dysfunction. (A) Tumour necrosis factor (pg/mL). (B) Interleukin-6 (pg/mL). (C) Interleukin-1β (pg/mL). (D) Interferon-gamma (pg/mL). A *t* test was used for statistical analysis. Graphs represent median and interquartile range. Dots and asterisks represent outliers. TNF - tumour necrosis factor; PGD - primary graft dysfunction; IL-6 - interleukin-6; IL-1β - Interleukin-1β; INF - interferon.

Primary graft dysfunction occurred in 52.6% of the transplant recipients. To analyze donor characteristics potentially related to PGD, we divided recipients into two groups: group 1, patients who developed PGD; and group 2, patients who did not develop PGD. Blood samples were obtained from donors with a median of 12 hours (10 - 18 hours), and the plasma cytokine values were then compared between the two groups of transplant recipients to evaluate the effects of BD-induced inflammatory activity on the outcome of transplants. The results were as follows: TNF, group 1: 10.8 (4.3 - 30.8) *versus* group 2: 8.7 (4.1 - 33.1) pg/mL, p = 0.63; IL-6, group 1: 1617.8 (106.7 - 5361.7) *versus* group 2: 922.9 (161.7 - 5361.7) pg/mL, p = 0.56; IL-1β, group 1: 0.1 (0.1 - 126.1) *versus* group 2: 0.1 (0.1 - 243.6) pg/mL, p = 0.60; and IFN-γ, group 1: 0.03 (0.02 - 0.2) *versus* group 2: 0.03 (0.02 - 0.1) pg/mL, p = 0.93). Donor plasma cytokine values did not differ between groups (Figure 1).

When kidney transplants were analyzed separately (n=28), donor age (group 1: 58 ± 9.6 *versus* group 2: 48 ± 13.1 years, p = 0.035), duration of ventilatory support (group 1: 96 [48 - 240] *versus* group 2: 48 [9 - 114] hours, p = 0.003), and length of hospital stay before BD diagnosis (group 1: 5.5 [2 - 14] *versus* group 2: 2 [1 - 9] hours, p = 0.019) were different between the two groups, but plasma sodium (group 1: 156 ± 8.4 *versus* group 2: 158 ± 12.4mEq/L, p = 0.6) and final creatinine (group 1: 1.3 ± 0.6 *versus* group 2: 1.3 ± 0.4mg/dL, p = 0.6) were not different. Additionally, plasma cytokine levels were not different between groups: TNF, group 1: 10.8 (4.3-30.8) *versus* group 2: 15.5 (5.2 - 33.0) pg/mL, p = 0.32; IL-6, group 1: 2312.7 (106-5361.7) *versus* group 2: 922.9 (161.7 - 5000) pg/mL, p = 0.63; IL-1β, group 1: 0.1 (0.1 - 126.1) *versus* group 2: 0.1 (0.1 - 243.6) pg/mL, p = 0.70; and IFN-γ, group 1: 0.04 (0.02 - 0.21) *versus* group 2: 0.06 (0.02 - 0.11) pg/mL, p = 0.29. The rate of delayed graft function (DGF) in kidney transplant recipients was 60.7%.

Subsequently, we divided patients into two groups, above and below cytokine median values (median TNF = 9.8pg/mL; median IL-6 = 923pg/mL; median IL-1β = 0.1pg/mL; and median IFN-γ = 0.04pg/mL) and we tested the association with PGD development. However, no association was detected (TNF, p = 0.63; IL-6, p = 0.59; IL-1β, p = 0.50; and IFN-γ, p = 0.85). Additionally, we used 193pg/mL as a cut-off point for IL-6 but found no differences in PGD development (p = 0.62).

In a logistic regression model with PGD as the dependent factor and donor age, duration of ventilatory support, cold ischemia time, TNF, and IL-6 as cofactors, cold ischemia time was the only variable associated with PGD development (odds ratio - OR = 0.85, 95% confidence interval - 95%CI 0.74 - 0.98, p = 0.032).

We also tested correlations between donor plasma cytokines and clinical variables. There was a moderate positive correlation between TNF, IL-6, and IL-1β and donor age (TNF: r = 0.35 p = 0.021; IL-6: r = 0.45, p = 0.002; and IL-1β: r = 0.41, p = 0.006). Additionally, a moderate positive correlation was found between plasma sodium levels and TNF (r = 0.36, p = 0.018), but there was no correlation with the other cytokines (IL-6: r = 0.28, p = 0.07; IL-1β: r = -0.16, p = 0.29; and IFN-γ: r = 0.12, p = 0.1).

## DISCUSSION

In this sample of recipients of organs from deceased donors, BD-induced plasma cytokine release was not associated with PGD, as donor plasma TNF, IL-6, IL-1β, and IFN-γ levels did not differ between transplant recipients who developed PGD and those who did not.

Primary graft dysfunction is a serious complication of transplantation that results from ischemia-reperfusion injury, a process triggered by the systemic inflammatory state of the donor.^(^^20^^)^ Patients who died within 30 days of lung transplant had elevated levels of IL-6 in biopsies prior to implantation.^(^^21^^)^ Likewise, myocardial TNF mRNA expression during organ retrieval predicted right ventricular dysfunction in heart recipients.^(^^8^^)^ Moreover, liver biopsies from deceased donors showed higher CD4 and CD8 infiltration than biopsies from living donors.^(^^22^^)^ Taken together, these studies suggest an association between increased organ tissue inflammation and PGD. However, our study did not show an association between plasma cytokine levels and PGD. One possible explanation for the lack of association between systemic cytokine levels and PGD, in contrast with the findings reported for tissue levels, is that the degree of tissue inflammation is higher than that measured in plasma, suggesting that tissue inflammation levels might be a better predictor of PGD than plasma inflammation levels. Furthermore, the high rate of DGF in kidney recipients (60.7%) observed in our sample of patients, which is consistent with that reported in another Brazilian study,^(^^11^^)^ might have prevented us from detecting a difference between groups.

Murugan et al. recruited 30 deceased donors and analyzed the outcomes of the respective 78 transplant recipients. In this cohort of patients, higher plasma IL-6 levels but not TNF and IL-10 levels before organ procurement correlated with a trend to lower 6-month hospital-free survival in recipients.^(^^19^^)^ Therefore, we used the cut-off value of IL-6 suggested in their study (193pg/mL), but no association with PGD was found for values above or below this threshold, suggesting that plasma IL-6 may not be a good predictor of early outcomes, such as PGD. In the short term, cold ischemia time and donor age appeared to be better predictors of outcome, as demonstrated previously^(^^11,23,24^^)^ and supported by our findings.

In a previous study, we demonstrated an upregulation of plasma TNF and IL-6 in deceased donors compared to controls.^(^^4^^)^ Interestingly, in the study by Murugan et al., plasma concentrations of IL-6 immediately before organ procurement were lower in donors treated with corticosteroids than in untreated donors.^(^^19^^)^ Likewise, Kotsch et al., in a randomized controlled trial, showed that methylprednisolone therapy in deceased donors reduces inflammation in the donor liver and improves outcome after liver transplantation.^(^^25^^)^ However, we did not find an association between plasma cytokine levels and liver PGD.

Elevated plasma IL-6 levels have been associated with a poorer prognosis in a variety of critical care settings^(^^26-28^^)^ and with lower organ yield in transplantation settings.^(^^19^^)^ However, to the best of our knowledge, this study is the first to evaluate donor plasma TNF, IL-6, IL-1β, and IFN-γ levels as predictors of PGD development in organs transplanted from brain-dead donors. This study had several limitations. First, the sample size was calculated to detect a difference of one SD in TNF log between brain-dead and control patients without brain-dead in a previous study^(^^4^^)^ and, in fact, may be underpowered to detect differences in PDG development between groups of transplant recipients. However, when estimating the sample size required to detect a difference in the present study, a sample size of at least 770 organ transplant recipients would be necessary. Second, we measured plasma cytokines only at the time of organ procurement, which may have been late in the inflammatory process, as cytokines peak earlier after BD. Third, we believe that a time course with earlier time points, such as 1, 2 and 6 hours, and up to 12 hours, might provide more consistent information about inflammation in brain-dead donors and its association with PGD development.

## CONCLUSION

Primary graft dysfunction is a predictor of worse short- and long-term outcomes after transplantation. In this respect, detecting clinical and laboratory variables that can accurately predict the development of primary graft dysfunction would be clinically relevant. Plasma cytokines can be easily and quickly measured, but the small sample size and the single time point measurement of plasma cytokines in our study preclude a conclusion regarding the association of donor plasma TNF, IL-6, IL-1β, and IFN-γ values with development of primary graft dysfunction. Then, the role of inflammatory cytokines, as a possible pathway associated with primary graft dysfunction development, should be the focus of investigation of larger studies.
